# Validation study of villous atrophy and small intestinal inflammation in Swedish biopsy registers

**DOI:** 10.1186/1471-230X-9-19

**Published:** 2009-03-11

**Authors:** Jonas F Ludvigsson, Lena Brandt, Scott M Montgomery, Fredrik Granath, Anders Ekbom

**Affiliations:** 1 Department of Pediatrics, Örebro University Hospital, Örebro, Sweden; 2 Clinical Epidemiology Unit, Karolinska University Hospital, Karolinska Institutet, Stockholm, Sweden; 3 Clinical Research Center, Örebro University Hospital, Örebro, Sweden; 4 Department of Primary Care and Social Medicine, Charing Cross Hospital, Imperial College, London, UK

## Abstract

**Background:**

Small intestinal biopsy with villous atrophy (VA) is the gold standard for the diagnosis of celiac disease (CD). We validated VA (Marsh 3) and small intestinal inflammation without VA (Marsh 1+2) in Swedish regional biopsy registers.

**Methods:**

All pathology departments in Sweden (n = 28) were searched to identify individuals with VA or duodenal/jejunal inflammation. The validation consisted of blinded examination of biopsy samples, manual review of biopsy reports, web surveys, and patient chart reviews of 121 individuals with VA and 39 with inflammation.

**Results:**

We identified 29,148 individuals with VA and 13,446 individuals with inflammation. In a blinded examination, Swedish pathologists correctly classified 90% of biopsies with VA. Manual screening of 1,534 biopsy reports (performed by co-author JFL and a research assistant) found that comorbidity other than CD was rare. IBD was the most common comorbidity and occurred in 0.3% of biopsies with VA (1.6% in inflammation). Among 114 patients with VA and available data, 108 (95%) had a clinical diagnosis of CD. 79% of the validated individuals with VA and 64% of those with inflammation had documented gastrointestinal symptoms prior to biopsy. 88% of the validated individuals with VA had positive CD serology before their first biopsy. 172/180 (96%) of Swedish gastroenterologists and 68/68 (100%) of pediatricians perform a small intestinal biopsy in at least 9 out of 10 individuals prior to diagnosis of CD.

**Conclusion:**

Regional biopsy data are feasible to identify individuals with CD and small-intestinal inflammation. The specificity of CD is high in villous atrophy.

## Background

**Figure 1:**
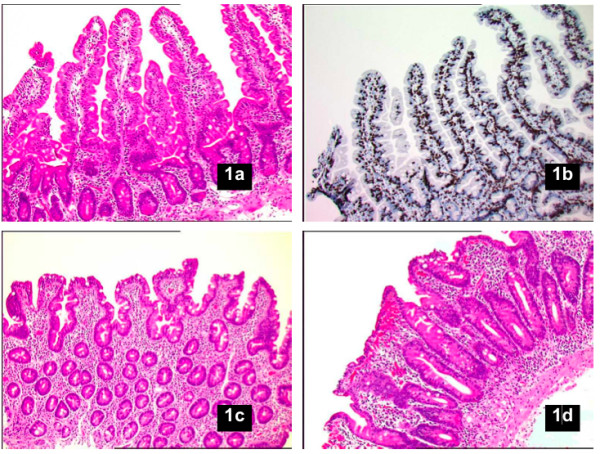
**Photographs of the small intestine: classifcation of histopathology**. a. Normal mucosa. b. Inflammation (intraepithelial lymphocytosis). c. Partial VA (CD). d. Total VA (CD). (Acknowledgement. Photomicrographs obtained from Prof. Åke Öst, earlier chairman of the Swedish National Steering Group for Small Intestinal Pathology).

Small intestinal biopsy is the gold standard of celiac disease (CD) diagnosis [1]. In a patient with CD, the biopsy typically shows villous atrophy (VA; Figure 1). Treatment with a gluten-free diet should then lead to improvement in symptoms and intestinal morphology on control biopsy [2,3].

We identified 29,148 individuals with VA and 13,446 individuals with small intestinal inflammation (called "inflammation" in this paper) through a search of all pathology departments of Sweden (n = 28).

The overall aim of the current study was to validate VA and inflammation in small intestinal biopsies obtained from Swedish biopsy registers and to evaluate if biopsy registers can be used as a data source for CD and inflammation. We specifically examined a) the accuracy of histopathological classification of small intestinal biopsies by Swedish pathologists; b) the proportion of biopsy reports with VA or inflammation where other disease than CD is mentioned; c) to what extent VA corresponds to a clinical diagnosis of CD; d) the characteristics (symptoms and CD serology) of 160 validated individuals with VA or inflammation; and e) the use of small-intestinal biopsy among Swedish gastroenterologists and pediatricians to diagnose CD.

## Methods

### Classification of biopsy data

Pathologists in Sweden, and elsewhere, will routinely examine the presence (or absence) of VA, crypts, the different layers of the intestine, and the number of intraepithelial lymphocytes (IELs) (Table 1) in small intestinal biopsies. Finally, the pathologist examines the brush border for evidence of infection. In Sweden, all small intestinal biopsies are classified according to the SnoMed system for diagnostic and register purposes. *SnoMed *("Systematized Nomenclature of Medicine-Clinical Terms") is a multilingual clinical healthcare terminology http://www.ihtsdo.org/our-standards/. In Sweden, SnoMed codes are often complemented with a "KVAST/Alexander" code" that is communicated to the physician who performed the biopsy. From an international perspective, the most common classification system of CD is the Marsh system ("Marsh-Oberhuber") [4], although recently Corazza et al suggested an alternative classification system with improved degree of interobserver reproducibility [5]. Table 1 contains an overview of different histopathology classifications and lists the SnoMed codes used in this project to identify individuals with VA and inflammation. In this project, inflammation is equivalent to Marsh 1+2 and VA equivalent to Marsh 3. Swedish SnoMed classification does not allow distinction between Marsh 1 and Marsh 2.

**Table 1:** Small intestinal histopathology classifications – a comparison

*Classification used in this project*	*Normal*	*Inflammation*		*Villous atrophy*		
Marsh Classification*	Type 0	Type 1	Type 2	Type 3a	Type 3b	Type 3c

Marsh Description	Pre-infitrative	Infiltrative	Infiltrative-hyperplastic	Flat destructive		

Corazza et al [5]	-	Grade A		Grade B1		Grade B2

SnoMed Codes	M0010, M0011	M40000, M41000, M42000, M43000, M47000, M47170		M58, D6218, M58005	M58, D6218, M58006	M58, D6218, M58007

KVAST/Alexander classification	I Normal	II Intraepithelial lymphocytosis (IEL)#		III Partial VA	IV Subtotal VA	IV Total VA

						

*Characteristics*						

Villous atrophy	-	-	-	+	++	++

IEL#	-	+	+	+	+	+

Crypt hyperplasia	-	-	+	+	++	++

*We have not included Marsh type 4 in this classification since such lesions are very rare [9] and cannot be identified through SnoMed Codes.

# Increased intraepithelial lymphocyte count (often >30/100 epithelial cells).

### Collection of biopsy data

Biopsy data were collected through computerized searches of all regional pathology departments in Sweden (28 out of 28 departments). These departments also included the private pathology department Medilab, which is responsible for the examination of biopsy samples from many private pathologists, as well as samples from the Gotland County. Medilab has also archived biopsy results from former private pathologists who have retired. Regional biopsy data in Sweden are primarily archived in two different computer systems "Safir" and "Sympathy". Local IT technicians were asked to perform searches for appropriate morphology codes and then save data on a) arrival date of the biopsy, b) Personal Identity Number, c) morphology and d) topography (T64 = duodenum; T65 = jejunum). Computerized searches were then exported to Microsoft Excel format and delivered to the researchers. In total we received 189 Microsoft Excel files from the 28 pathology departments. Each file was examined by JFL and LB. On one occasion, the computerized search was inadequate and had to be re-performed at the regional pathology department. JFL and LB then collaborated on cleaning the data and identifying data irregularities. Biopsy data were finally merged into one file.

Initially we collected all small intestinal biopsy samples (normal mucosa, inflammation and VA) and at the end of the data collection, identified individuals with VA and inflammation according to relevant SnoMed codes. Data collection took place between the 27^th ^of October 2006 and the 12^th ^of February 2008.

### Validation of VA and inflammation

*The accuracy of histopathological classification of small intestinal biopsies *was surveyed in 1999, when the Swedish National Steering Group for Small Intestinal Pathology sent out small intestinal biopsy samples to 22 Swedish pathology departments. Each biopsy sample had first been categorized through consensus of the steering group (gold standard) and was then graded by blinded pathologists from the 22 pathology departments. This validation (distribution of biopsy samples and collection of results) was coordinated by the non-profit company Equalis http://www.equalis.se/. Data on the accuracy were obtained from Equalis and compiled by JFL.

We evaluated the specificity of VA and inflammation. We did so by *estimating the proportion of biopsy reports (with VA or inflammation) where other disease than CD was mentioned*. In total we had data on 56,176 unique biopsies (VA: 38,655; inflammation: 17,521) in 29,148 individuals with CD and 13,446 individuals with inflammation. A computerized search of the plain text of these biopsy reports yielded 1,534 biopsy reports where there were indications of comorbidity other than CD. Each of these biopsy reports (n = 1,534) was then manually screened by two independent reviewers (JFL and research assistant) to confirm or reject the presence of comorbidity. Discrepancies were resolved through a third review of the biopsy report in question.

The manual review of biopsy reports was then complemented with a *review of patient charts *from 160 individuals undergoing small intestinal biopsy (121 with VA and 39 with inflammation). These individuals were randomly selected from five counties contributing biopsy data (these counties in total supplied 5,317 individuals with VA and 2,215 with inflammation). These 160 validated individuals had been referred for biopsy from 45 different clinics or health care centers. We specifically looked at the clinical characteristics of patients with VA and inflammation and dietary treatment and dietary compliance in individuals with VA. We were able to characterize 118 individuals with VA and 39 individuals with inflammation with regards to clinical symptoms and laboratory measures. There were patient chart data on dietary compliance in 71–101 individuals with VA. Finally we obtained all available CD serology data on the 160 validated individuals described above in order to correlate CD serology with VA.

We also examined the clinical management of CD in Sweden. This included the use of small intestinal biopsy prior to diagnosis of CD, the histopathological examination of small intestinal biopsies by pathologists and the treatment of CD. For this purpose we carried out three web surveys. Potential survey respondents were identified through member registers, email lists and through the Swedish Association of Pathology.

To identify (adult) gastroenterologists, we matched the member register of the Swedish Gastroenterology association with the administrative register of Swedish physicians http://www.hsar.se/ and identified 282 active gastroenterologists with a postal address in Sweden and an e-mail address. These individuals were then sent information by post and email about a survey on CD in adults, as well as the web address of an 11-item-survey. The results of this survey have previously been published in a Swedish-language journal (*Gastrokuriren*).

We identified pediatricians with an interest in gastroenterology through two existing e-mail lists for Swedish pediatricians (one for CD and one for general pediatric gastroenterology, including IBD). 70 pediatricians were then emailed information about a survey on CD in children, as well as the web address of a pediatric 11-item-survey. The results of this survey have previously been published in a Swedish-language journal (*Barnläkaren*).

After permission from the Swedish-language journals *Gastrokuriren *and *Barnläkaren*, we included data on CD investigation and management in this paper[6,7], since these Swedish journals are not indexed by Medline (or any other medical database) and contain no English-language abstract. Written permissions from *Gastrokuriren *and *Barnläkaren *have been separately uploaded to *BMC Gastroenterology*.

To define the histopathological examination of small intestinal biopsies, we emailed 25 pathology departments and asked them to answer an 18-item-survey. Although, 28 pathology departments contributed data to our study, 3 smaller pathology departments participating in our study formally had the same head pathologist as the university hospital of their health care region and referred us to the larger department for our survey.

### Statistics

We calculated 95% confidence intervals (CI).

### Ethics

The current study was performed as part of a larger project on complications in CD and inflammation. That study was approved by the Regional Ethical Review Board in Stockholm on the 4^th ^of June 2006 (2006/633-31/4) with additional amendments (2007/747-32 and 2008/257-32).

For the validation of the 160 patients, we obtained specific permission from the National Board of Health and Welfare.

## Results

We identified 381,043 small intestinal biopsies. Of these, 28,654 were duplicates (biopsies could be identified more than once if histopathology SnoMed codes indicated both inflammation and villous atrophy). Of the remaining 352,389 biopsies we excluded 986 due to data inconsistencies (including incorrect Personal Identity Number). The final 351,403 small intestinal biopsies, from which we identified individuals with VA and inflammation, represented 287,586 unique individuals. 240,922 individuals had only one small intestinal biopsy. Less than 1% had been biopsied on more than three occasions.

We identified 29,148 unique individuals with VA at some stage and 13,446 unique individuals with inflammation at some stage but never VA. The median year of biopsy with VA or inflammation was 1998, ranging from 1969–2008 in individuals with VA to 1970–2008 in individuals with inflammation. Except for the above description of the biopsy data collection, all data in this paper refer only to individuals with VA or inflammation.

### Age and sex of individuals with a small intestinal biopsy

The majority of study participants were females; and adults predominated at first biopsy (Table 2). In individuals with VA 36.8% were children (≤ 15 years) at first positive biopsy, compared with 6.1% of those with inflammation.

**Table 2:** Characteristics of unique individuals at first positive small intestinal biopsy

	*VA*	*Inflammation*
Number	29,148	13,446

Age, yrs (median, range)	30; 0–102	48; 0–104

Children ≤ 15 years (%)	10,718 (36.8)*	821 (6.1)#

Children ≤ 21 years (%)	12,273 (42.1)	1,515 (11.3)

Females (%)	18,033 (61.9)	7,575 (56.3)

Entry year (median, range)	1998; 1969–2008	1998; 1970–2008

*Only includes biopsies with villous atrophy. 46 individuals with their first "positive biopsy" (characterized by villous atrophy) after the age of 15 years had had a biopsy *without *VA when aged ≤ 15 years. Including them, the proportion of individuals with villous atrophy having "any small intestinal biopsy" ≤ 15 years of age was 36.9%.

#Only includes biopsies with inflammation. 24 individuals with their first "positive biopsy" (characterized by inflammation without villous atrophy) after the age of 15 years had had a normal biopsy when aged ≤ 15 years. Including them, the proportion of individuals with inflammation having "any small intestinal biopsy" ≤ 15 years of age was 6.3%.

### Validation of VA and inflammation

#### Blinded classification of small intestinal biopsies

Ninety percent (90%; 95% CI = 87–94%) of biopsies with VA were correctly classified as defined by the National Steering Group of Small Intestinal Pathology (Table 3). The proportions of missing data were independent of the grading of the biopsy. Fifty-six percent (41–72%) of biopsies with inflammation were correctly classified.

**Table 3:** Accuracy of histopathological classification

	*Samples mailed* *to pathologists*	*Not* *Responding*	*Correctly* *classified*	*Worst case* *Scenario#*
Normal*	84	7 (8%)	74/77 (96%)	74/84 (88%)

Inflammation*	42	3 (7%)	22/39 (56%)§	22/42 (52%)

Villous atrophy*	231	21 (9%)	190/210 (90%)	190/231 (82%)

* According to gold standard (= grading by the Swedish National Steering Group for Small Intestinal Pathology).

# Assuming that all missing responses were incorrect.

§ Of the misclassified 17 samples with inflammation (according to the steering group, i.e. gold standard), 12 were classified as normal, and 5 as villous atrophy.

#### Manual examination of biopsy reports

Our examination of 1,534 biopsy reports, found that small intestinal pathology other than CD, as mentioned in the biopsy report, was uncommon in VA or inflammation (Table 4). The most common comorbidity was IBD, occurring in 0.3% (0.2–0.3%) of biopsies with VA and 1.6% (1.4–1.7%) of biopsies with inflammation (see Additional File 1.

**Table 4:** Comorbidity in biopsy samples – results of manual examinations of 1,534 biopsy reports

*Histopathology*	*Villous Atrophy*	*Inflammation*
***Samples, No***.	38,655 (%*)	17,521 (%*)

		

Autoimmune enteropathy	10	21 (0.1)

Gastric metaplasia	37	186 (1.1)

Giardiasis	5	2

Helicobacter pylori	73 (0.2)	70 (0.4)

Inflammatory granuloma	14	41 (0.2)

Lymphoma	13	7

Cancer other than lymphoma	29	51 (0.3)

Tropical sprue	1	0

Crohn's disease	21	94 (0.5)

Colitis: Microscopic/Ulcerative	73 (0.2)	163 (0.9)

Any IBD#	98 (0.3)	272 (1.6)

Postoperative changes	17	60 (0.3)

Vasculitis	1	0

Refractory CD	3	0

We also searched plain biopsy text for immune deficiency, Behcet's disease, graft vs. host disease/transplantation, sarcoidosis, immunoglobuline disease, Systemic Lupus Erythematosus, Whipple's disease and Zollinger-Ellison Disease. For these disorders, the computerized search only yielded results that were rejected on manual examination.

*Percentages are listed where the prevalence is 0.1% or more.

#Includes biopsy reports where non-specific IBD was listed.

IBD = Inflammatory bowel disease

#### CD among individuals with VA

**Figure 2:**
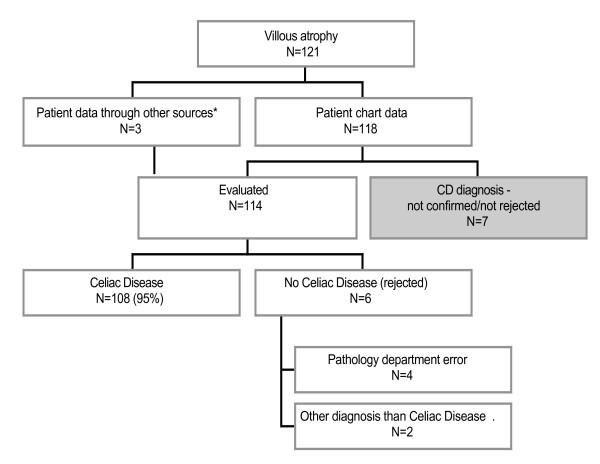
**Overview of patient chart review**. 95% of all individuals with VA had a clinical diagnosis of CD according to the patient charts (95% CI = 91–99%). This is consistent with data from an earlier Swedish study showing that out of 524 children with a positive first biopsy, 509 improved in their mucosa when starting on a gluten-free diet (corresponds to 97.1%)[8]. *Other sources: In two patients, we phoned the responsible physician and the diagnosis of CD could then be confirmed. In a third patient, no patient chart could be obtained. However, through the local chemistry laboratory, we obtained data on CD serology that showed positive endomysium and antigliadin antibodies prior to biopsy, and negative endomysium and antigliadin antibodies after biopsy. Also this patient was regarded as having CD.

According to patient chart data, VA had a high specificity for CD. 108 (95%; 91–99%) out of 114 patients with VA and available data had a clinical diagnosis of CD (Figure 2) [8]. In seven individuals (121 minus 114), the CD diagnosis could neither be confirmed, nor rejected (Figure 2). This was due to emigration, inconsistent SnoMed coding etc. In individuals where the diagnosis was rejected (n = 6), four biopsies (patients 1–4) had been misclassified in the relevant department of pathology. Patient 5 had VA, but received gluten-containing food after the diagnostic biopsy. A second upper endoscopy 18 years later found that patient 5 had a normal mucosa and the CD diagnosis was re-evaluated. In patient 6, a biopsy initially classified as "VA" was later re-examined (on request from the physician) and then found not to fulfill the criteria for VA (or CD).

#### Clinical characteristics of individuals with VA and inflammation

Clinical characteristics of the reviewed individuals are presented in Table 5. Most individuals presented with classical features, with diarrhea being the most common symptom in individuals with VA. Although, 64% (49–79%) of individuals with inflammation had some gastrointestinal symptom prior to biopsy, the single most common symptom/sign was anemia (31%; 16–45%). Folic acid deficiency was reported in 22% (15–30%) of individuals with VA, but only in 5% (1–17%) of individuals with inflammation.

**Table 5:** Clinical characteristics of individuals with VA and inflammation – patient chart review.

	*Villous atrophy;* *N *= *118*	*Inflammation;* *N *= *39*
*Background data*		

Females	72 (61)	16 (41)

Median age at first biopsy; range (years)	42; 1–86	53; 1–84

Median follow-up; range (years)	7; 0–24	6; 0–17

*Heredity*		

Reported heredity for CD	14 (12)	2 (5)

Reported heredity for type 1 diabetes	3 (3)	0 (0)

*Other diseases*		

Diabetes Mellitus, type 1	5 (4)	1 (3)

Dermatitis herpetiformis	6 (5)	0 (0)

Other autoimmune disease	11 (9)	8 (21)

*Symptoms*		

Any gastrointestinal symptom*	93 (79)	25 (64)

Diarrhea	42 (36)	7 (18)

Anemia	41 (35)	12 (31)

Weight loss/growth failure	34 (29)	3 (8)

Abdominal pain	22 (19)	9 (23)

Vomiting or nausea	15 (13)	6 (15)

Constipation	12 (10)	1 (3)

Fatigue	9 (8)	3 (8)

*Laboratory data*		

Iron-deficiency	32 (27)	5 (13)

Folic acid deficiency	26 (22)	2 (5)

B12-deficiency	16 (14)	1 (3)

Thrombocytes, increased	15 (13)	4 (10)

Low albumin	12 (10)	7 (18)

Erythrocyte sedimentation rate, increased	9 (8)	3 (8)

Liver enzymes, increased	9 (8)	4 (10)

*Biopsy report*		

Original biopsy report could be examined through patient chart reviews	98 (83)	33 (85)

Clinical data (about the patient) is described in the biopsy referral letter (to the pathologist)#	90 (92)	28 (85)

Macroscopic appearance of the intestine is described in the referral letter#	39 (40)	14 (42)

Percentages are given within brackets. Although, the diagnosis CD was evaluated in 121 individuals, patient chart data were only available in 118 (98%).

*Weight loss was included among "any GI symptom". Also reflux symptoms (not listed above) were included among "any GI symptoms".

#Based on 98 and 33 biopsy reports respectively.

93% of individuals with VA (94/101; 88–98%) had received dietary information according to patient chart data. There were indications of low dietary compliance (not strict gluten-free diet) in 15/86 individuals with VA (17%; 9–25%). Five out of 71 patients (7%; 1–13%) did not respond satisfactorily to a gluten-free diet. Two of the patients with little response to a gluten-free diet however admitted low dietary compliance.

Two individuals (5%; 1–17%) with inflammation (and who had never had VA) had at some stage received information about gluten-free diet. None of these individuals had however received a clinical diagnosis of CD according to the patient chart.

In 71 (88%; 80–95%) out of 81 individuals with VA and available CD serology data, there was at least one positive antibody test (gliadin, endomysium or transglutaminase) prior to first biopsy (Table 6). After biopsy, most serology samples in the validated individuals with VA were negative (Table 6). Among 39 individuals with *inflammation *(and no VA), two (5%; 1–17%) had a positive CD serology prior to biopsy (none of them received a clinical diagnosis of CD according to patient charts).

**Table 6:** CD serology before and after first biopsy in individuals with VA

*Positive serology before biopsy (%)*			*Negative serology after biopsy (%)*		
					

Gliadin	Endomysium	Transglutam.	Gliadin	Endomysium	Transglutam.

39/53 (74)	46/56 (82)	28/33 (85)	54/60 (90)	31/36 (87)	29/35 (83)

#### Clinical management of CD

Our web surveys found that most gastroenterologists and pediatricians biopsy their patients prior to CD diagnosis and that almost all patients with CD receive information about gluten free diet.

Among 282 available gastroenterologists, 184 (65%) gastroenterologists participated in our survey. 96% (91–98%) of Swedish gastroenterologists perform a small intestinal biopsy in at least 9 out of 10 patients prior to diagnosis of CD (Table 7). 93% (89–97%) inform at least 9 out of 10 patients with CD about gluten-free diet.

68 pediatricians (97% of those contacted) participated in our survey (Table 8). 100% (95–100%) of the pediatricians perform a small intestinal biopsy in at least 9 out of 10 patients prior to diagnosis of CD. 97% (90–100%) inform at least 9 out of 10 patients with CD about gluten-free diet.

**Table 7:** Management of CD among gastroenterologists

*Topic*	≥ *9 out of 10 patients*	*5–8/10 patients*	*1–4/10 patients*	*No patient/* *never*	Total*
Biopsy performed prior to CD diagnosis	172 (96)	7 (4)	0 (0)	1 (0.6)	180

Control biopsy performed to verify mucosal healing on gluten-free diet	64 (36)	29 (16)	57 (32)	30 (17)	180

Gluten provocation and third biopsy performed	1 (0.6)	1 (0.6)	16 (9)	161 (90)	179

CD serology is part of "my diagnostic algorithm for CD"	141 (78)	14 (8)	19 (11)	6 (3)	180

"My patients with CD" receive information about gluten-free diet by health-care personnel	164 (93)	1 (0.6)	4 (2)	7 (4)	176

*Number of respondents (percentages within brackets were calculated based on these numbers).

Table 7 has previously been published in the Swedish-language journal *Gastrokuriren *[6,7], and is re-published after permission from that journal.

**Table 8:** Management of CD among pediatricians

*Topic*	≥ *9 out of 10 patients*	*5–8/10 patients*	*1–4/10 patients*	*No patient/never*	Total*
Biopsy performed prior to CD diagnosis	68 (100)	0 (0)	0 (0)	0 (0)	68

Control biopsy to verify mucosal healing on gluten-free diet	9 (14)	3 (5)	36 (55)	18 (27)	66

Gluten provocation and third biopsy performed	0 (0)	0 (0)	38 (59)	26 (41)	64

CD serology is part of "my diagnostic algorithm for CD"	68 (100)	0 (0)	0 (0)	0 (0)	68

"My patients with CD" receive information about gluten-free diet by health-care personnel	65 (97)	0 (0)	0 (0)	2 (3)	67

*Number of respondents (percentages within brackets were calculated based on these numbers).

Table 8 has previously been published in the Swedish-language journal *Barnläkaren *[6,7], and is re-published after permission from that journal.

#### Survey of pathology departments: evaluation of CD and inflammation

Among 25 contacted departments of pathology, 23 (92%) responded to our histopathology survey. 20/23 (87%; 73–100%) pathology departments use a Swedish histopathological classification system called "KVAST/Alexander", with 3 departments using the Marsh classification when they communicate pathology findings to the physician (Table 1). All pathology departments however also classify biopsy samples according to the international SnoMed classification system http://www.snomed.org/. 20/23 (87%; 73–100%) pathology departments reported that most pediatricians and gastroenterologists would send 2–3 tissue samples from the duodenum/jejunum (originating from one endoscopy session) for referral (1 (4%) reported that most gastroenterologist would send only 1 biopsy samples for microscopic examination; while 2 departments (9%) reported 4 or more samples to be routine). 19/23 (83%; 67–98%) pathology departments said that their histopathology report would be based on the most severe finding at microscopic examination, while 4 (17%) reported that the most common finding would form the basis of their pathology report. When asked what (minimum) villous-crypt ratio would be accepted as normal, 3:1 was used in most departments (18/23; 78%; 64–95%) (all other departments reported a 4:1 (13%) or a 2:1 (9%) ratio to be accepted as normal). None of the pathology departments requested a 5:1 ratio. 22/22 (100%; 85–100%) pathology departments used immunostaining for CD3 to detect IELs. 19/23 (83%; 67–98%) reported using 30 IELs per 100 as their cut-off for "increased IEL count", while 1 department reported using 25 and 3 departments used 20 as their cut-off. Other results of this survey are presented in Table 9.

**Table 9:** Management of CD in pathology departments (%)

*Topic*	≥ *9 out of 10 patients*	*5–8/10 patients*	*1–4/10 patients*	*No patients/never*	Total*
The referral note from the physician contains clinical information	18 (78)	4 (17)	1 (4)	0 (0)	23

The referral note from the physician contains data on macroscopic appearance of the small intestine	7 (30)	8 (34)	6 (26)	2 (9)	23

Suppose that a pathologist is uncertain of the grading of a small intestinal biopsy samples. How often will he/she ask for a second opinion?	5 (23)	5 (23)	8 (36)	4 (18)	22

* Number of respondents (percentages within brackets were calculated based on these numbers).

## Discussion

This paper describes the validation of VA and inflammation in small intestinal biopsies from the 28 pathology departments in Sweden. In Sweden, pathologists have traditionally divided CD-like lesions into a) VA and b) inflammation without VA, with further subdivisions into partial, subtotal and total VA. Inflammation (Table 1) corresponds to both Marsh 1 and Marsh 2 and grade A in the newly introduced classification by Corazza et al [5]. This means that Swedish biopsy registers do not distinguish between Marsh 1 and Marsh 2.

We identified 29,148 individuals with VA and 13,446 individuals with inflammation through computerized searches of all pathology departments in Sweden. In our validation, VA had a high specificity for CD, which is not surprising since other causes of VA than CD are rare in Western countries [9]. In our manual review of more than 1,500 biopsy reports, comorbidity such as IBD, giardiasis, helicobacter pylori-infection, gastric metaplasia or cancer was rare in VA or small intestinal inflammation (Table 4) (also for biopsy samples with inflammation, was other co-morbidity than CD uncommon. In "inflammation", IBD accounted for 1.6% of biopsies with small-intestinal inflammation). Our patient chart review found that 95% of biopsy reports with VA were consistent with clinical CD. Although, VA may be patchy and biopsy samples without atrophy may be misinterpreted as atrophic by the pathologist if the samples are wrongly oriented, earlier validation has shown that the vast majority (90%) of intestinal biopsies with VA are correctly classified by Swedish pathologists (Table 3). Although, not all pathologists responded to this survey (Table 3), missing responses were equally distributed between biopsies with normal mucosa, inflammation and VA. This suggests that missing responses were not biased towards biopsy samples that are difficult to evaluate since we then would have expected a higher rate of missing values in samples with inflammation. Pathologists also reported that almost 9 out of 10 pediatricians/gastroenterologists submit at least two biopsy samples from the small intestine (instead of just one sample) when CD is investigated. Multiple samples increase the likelihood of identifying VA. We conclude that biopsy data with VA may be used as a data source for CD, although comorbidity may be underestimated when evaluated from biopsy reports since not all comorbidity is mentioned in biopsy reports.

Unfortunately we did not have access to the referral notes from the physician. Such notes often contain additional clinical information about the reason for biopsy. With access to referral note data it is possible that we could have distinguished individuals with inflammation biopsied due to suspected CD from those biopsied due to other cause such as IBD. To some extent this can be done through manual screening of the running biopsy text, but it is still likely that inflammation in Swedish biopsy registers is a heterogeneous concept representing both CD and other diseases.

The sensitivity of *diagnosed CD *should be close to 100% through biopsy registers, since a duodenal/jejunal biopsy with VA is gold standard for CD diagnosis in Sweden [10,11] (see also Tables 7 and 8). However, although data on CD management were obtained from almost all pediatricians surveyed, our response rate was lower among gastroenterologists investigating and treating adults (65%). This response rate is similar to that in a review by Asch et al (usually 5–6 out of 10 physicians respond to mailed surveys) [12]. Nevertheless, there is a risk of selection bias and we cannot exclude that our respondents were more interested in CD than the average Swedish gastroenterologist and that this influenced the results of our survey.

Until now, most large Swedish cohort studies of CD have used the Swedish National Hospital Discharge Register to identify patents with CD (e.g. [13-16]). Regional biopsy registers do however offer several advantages over the Hospital Discharge Register when it comes to identifying CD (and inflammation). Foremost among these advantages may be higher specificity and sensitivity for both CD and inflammation.

While the Swedish Hospital Discharge Register and other diagnostic registers, cannot be used to identify inflammation, biopsy registers constitute a means to identify tissue-verified inflammation. Data from the Swedish National Steering Group for Small Intestinal Pathology show that 56% of biopsy samples with inflammation are correctly classified. This is lower than for VA but may still be higher than when using the traditional Marsh classification system. Our data are in fact similar to those reported by Italian researchers using a simplified Marsh classification system [5]. The Italian researchers had earlier noted that the interobserver reproducibility was lower for Marsh 1 and Marsh 2 (especially for Marsh 2) [5] than for Marsh 3 (especially Marsh 3c). However, partial Kappa scores increased significantly when they merged Marsh 1 and Marsh 2 lesions into what they call "grade A". This concept (Grade A) is actually identical to "small intestinal inflammation" in Swedish biopsy registers. The traditional use of grading small intestinal inflammation into VA vs. inflammation (without VA) stems from the long-term use of VA as the basis of CD diagnosis in Sweden [2] Increasingly it has however been recognized that CD may be present in the absence of VA and that additional measures including genetic and immunological markers may be important in the investigation of CD ([17]. The current study had no data on either genetic or immunological markers. Previous research suggests that up to 40% of individuals with inflammation (as defined by increased IEL count) may suffer from gluten sensitive enteropathy [18], but as noted earlier our histopathology approach does not distinguish between CD-inflammation and non-CD-inflammation.

Another weakness of our methodological approach is that we have no data on the exact numbers and distribution of IELs in biopsies with inflammation. It has been suggested that a uniform distribution of IELs is increases the proportion of CD in patients with inflammation without VA [19]. The lack of exact number and distribution of IELs in biopsies with inflammation is a limitation of our study.

"Positive biopsy" for VA has been the recommended gold standard for CD for the last 30–40 years[2,20]. In contrast, CD serology has only been generally available in Sweden since the mid-1990s. This means that using biopsy registers to identify individuals with CD allows for substantially longer follow-up of patients than if using CD serology data to identify patients with CD. Even though the sensitivity of e.g. tissue transglutaminase autoantibodies is high in *total *VA, it decreases to around 70% in *partial *VA (also CD) and is even lower in *inflammation *[21]. CD serology has a high specificity [22] for CD and a high negative predictive value. CD serology is therefore well suited to rule out the presence of CD. In contrast, the positive predictive value of CD serology has been disappointing, which is not surprising given that 99% of all Westerners do *not *have CD [23,24] (only a minute decrease in specificity will then result in false-positive cases). In two recent papers, the positive predictive value of tissue transglutaminase antibodies varied between 29–76%[25,26]. For the above reasons we chose to identify CD and inflammation through biopsy reports instead of serological data.

Symptoms and signs vary in CD [27-29]. "Classical CD" presents with chronic diarrhea, abdominal pain, other gastrointestinal symptoms and weight loss/growth retardation [30] and is still common in children [27]. Increasingly however, CD is detected in children with non-classic symptoms, or through screening of first-degree relatives and high-risk individuals (e.g. those with type 1 diabetes) [31]). In adults, diarrhea is probably the dominating symptom (i.e. the classic form), although silent CD [30] is also common [32]. One of the limitations of biopsy registers, is that they contain no data on symptoms. We therefore characterized 121 patients with VA and 39 patients with inflammation through patient chart reviews and collection of CD serology data. Most of our patients with VA presented with classical symptoms (diarrhea was the most common symptoms). One in three patients with VA and inflammation suffered from anemia at time of biopsy. Our data on anemia and diarrhea in VA are thereby consistent with earlier findings in CD[27,30,33]. Heredity for CD was found in 12% of individuals with VA and in 5% of individuals with inflammation. Although, we do not know of any study examining the heredity for CD in individuals with inflammation without VA, Dube et al [24] recently compiled data on CD heredity, with a pooled prevalence of biopsy-verified CD of 16% among first-degree relatives to CD patients. It is also noteworthy that 88% of the 81 validated patients with available CD serology data and VA in our study had at least one positive CD serological marker prior to first biopsy. Although, most patients with VA had negative CD serology after first biopsy, not all did. Positive serology after biopsy may be explained by a number of factors including low compliance, serology performed only shortly after biopsy, or being performed at time of gluten provocation. In addition, a number of patients with VA may have persistently abnormal CD serology despite gluten-free diet.

Among patients with VA and available data, we also characterized dietary compliance. This review found indications of low dietary compliance in 17% of individuals with VA. This is consistent with earlier data [34-37], although it should be remembered that our data on dietary compliance were retrospectively collected through patient charts. Another weakness of our data on dietary compliance is that the follow-up differed between patients, as did the time between CD diagnosis and evaluation of dietary compliance. Finally, there is a risk that the basis of compliance assessment differed between physicians and dieticians; and that patients without information on dietary compliance differ from those *with *such information. Only 2/39 validated patients with inflammation in our study had received information about gluten-free diet and none of them had received a clinical diagnosis of CD by their physician. This is not surprising, given that Swedish patients with small-intestinal inflammation by tradition do *not *receive a gluten-free diet.

## Conclusion

In conclusion, we merged data from all 28 pathology departments in Sweden and identified 29,148 individuals with VA and 13,446 individuals with small-intestinal inflammation. Our validation shows that it is feasible to identify individuals with CD and small-intestinal inflammation using regional biopsy data. The specificity of CD is high in villous atrophy.

## Abbreviations

CD: celiac disease; IEL: intraepithelial lymphocytes; VA: villous atrophy.

## Competing interests

The authors declare that they have no competing interests.

## Authors' contributions

JFL designed the study, wrote the ethics application, collected the data, analyzed the statistics and wrote the manuscript. LB helped JFL manage the data and critically reviewed the manuscript. SM, FG and AE critically reviewed the manuscript and contributed to the study design.

## Pre-publication history

The pre-publication history for this paper can be accessed here:

http://www.biomedcentral.com/1471-230X/9/19/prepub

## Supplementary Material

Additional File 1 **Table S1. **Comorbidity defined by SnoMed codes in patients with inflammation or villous atrophy (VA) – a single centre evaluation. Click here for file
