# Supplementary material for: Validation study of villous atrophy and small intestinal inflammation in Swedish biopsy registers
Source: BMC Gastroenterol. 2009 Mar 11;9:19. doi: 10.1186/1471-230X-9-19 (PMC2664822; doi:10.1186/1471-230X-9-19)
Supplement: Additional File 1 — Table S1. Comorbidity defined by SnoMed codes in patients with inflammation or villous atrophy (VA) – a single centre evaluation. [file 1471-230X-9-19-S1.doc]

**Appendix**

**Supplementary Table 1.**

**Comorbidity defined by SnoMed codes in patients with inflammation or villous atrophy (VA) – a single centre evaluation.**

| **Diagnosis** | **SnoMed** | **Infl.**  **(n=204)*** | **Infl.**  **(n=199)*§** | **VA**  **N=1,606#** | **VA**  **N=1,380#§** |
| --- | --- | --- | --- | --- | --- |
| Adenocarcinoma | M814/82 | 2 (1.0) | 2 (1.0) | 0 | 1 (<0.1) |
| Gastric/intestinal metaplasia | M7333 | 0 | 0 | 1 (<0.1) | 1 (<0.1) |
| Helicobacter pylori | ME1370 | 0 | 0 | 0 | 1 (<0.1) |
| Inflammatory granuloma | M44 | 0 | 4 (2.0) | 1 (<0.1) | 6 (0.4) |
| Lymphoma | M95/96 | 1 (0.5) | 1 (0.5) | 1 (<0.1) | 6 (0.4) |
| Crohn’s disease | D6216 | 0 | 3 (1.5) | 0 | 2 (0.1) |
| Ulcerative colitis | D6255 | 0 | 0 | 0 | 0 |
| Postoperative changes | M18000 | 0 | 2 (1.0) | 1 (<0.1) | 2 (0.1) |
| Whipple’s disease | D0840 | 0 | 0 | 0 | 0 |
| Giardiasis | ME4416 | 0 | 0 | 0 | 0 |
| TOTAL | - | 3 (1.5) | 12 (6.0) | 4 (0.2) | 19 (1.4) |

Infl, Inflammation. VA, villous atrophy.

As opposed to the computerized text search for co-morbidity (Table 4 in the paper), Supplementary Table 1 (* columns) shows the number of biopsies with both a SnoMed Code signifying inflammation/villous atrophy and a SnoMed code for a Comorbidity/diagnosis other than CD. NB. Codes may not be listed in the running text of the biopsy report.

E.g. For example 0.5% of all *biopsy reports* with inflammation (1/204) listed the SnoMed code for lymphoma.

# VA = Villous atrophy

§ Presence of SnoMed codes from the duodenum/jejunum in unique individuals at some stage of life. E.g. For example 0.4% of *all individuals* with villous atrophy (6/1,380) had a SnoMed code for lymphoma at some stage of life (prior to, simultaneously of, or after the first biopsy with VA).

**Supplementary Table 2. Comparison between Hospital Discharge Register and Biopsy Registers.**

| ***Identification through Hospital Discharge Register*** | ***Identification through biopsy registers*** |
| --- | --- |
| Many individuals with CD are not admitted to hospital. Restricting a study to inpatients with CD therefore results in *low sensitivity for CD*. | Using biopsy registers will yield a *high sensitivity (close to 100%)* for CD since positive biopsy is mandatory for CD diagnosis in Sweden (see pilot work). |
| *Very low sensitivity for inflammation*. Non-specific gastrointestinal inflammation has no ICD-10 code. | High sensitivity for inflammation when patient has symptoms suggestive of CD. |
| *Risk of hospital admission may be associated with other comorbidity or low dietary compliance*. Therefore, CD inpatients may have more severe CD, and an increased risk of other co-morbidities compared to the average patient with CD. | Use of diagnostic biopsy is not influenced by comorbidity, or later dietary compliance. Individuals identified are characterized by average CD severity. |
| *Hospital admission may vary with age*. Young children and old adults with CD are more likely to be admitted to hospital. | Biopsy registers will identify all individuals with CD, irrespective of patient age, and sex. |
| Date of hospital admission may not correlate with date of diagnosis. The first inpatient diagnosis of CD is sometimes registered only years after the first biopsy and institution of a gluten-free diet. | Date of first biopsy correlates with the start of gluten-free diet. |

**The Swedish Personal Identity Number (PIN)**

The unique Personal Identity Number (PIN) is assigned to more than 99.9% of all Swedish residents and immigrants [20]. Every record in the national health registers (including regional biopsy registers) can be identified through a PIN, and since 1984 the PIN has been registered in more than 99% of all inpatient care reported to the National Board of Health and Welfare. Exceptions (and missing values) may consist of e.g. newborns that have not yet received a PIN, and women undergoing abortions (PIN is not registered in the national statistics on abortions due to integrity reasons). All Swedish government agencies use the PIN for register matching.

**EQUALIS**

Equalis (External Quality Assurance in Laboratory Medicine In Sweden) is jointly owned by Salar Enterprises, The Swedish Society of Medicine, and The Swedish Institute of Biomedical Laboratory Science, with initial support from the National Board of Health and Welfare. Equalis aims to increase patient safety by continuously improving the quality of laboratory medicine activities.
